# Radiomics Analysis of Multi-Phase DCE-MRI in Predicting Tumor Response to Neoadjuvant Therapy in Breast Cancer

**DOI:** 10.3390/diagnostics11112086

**Published:** 2021-11-11

**Authors:** Shuyi Peng, Leqing Chen, Juan Tao, Jie Liu, Wenying Zhu, Huan Liu, Fan Yang

**Affiliations:** 1 Department of Radiology, Union Hospital, Tongji Medical College, Huazhong University of Science and Technology, Wuhan 430022, China; shuyipeng@hust.edu.cn (S.P.); leqingchen@hust.edu.cn (L.C.); 2014xh0917@hust.edu.cn (J.T.); liu_jie0823@163.com (J.L.); zhuwenying917@163.com (W.Z.); 2 Hubei Province Key Laboratory of Molecular Imaging, Wuhan 430022, China; 3 Precision Healthcare Institute, GE Healthcare, Shanghai 201203, China; Huan.Liu@ge.com

**Keywords:** breast cancer, magnetic resonance imaging, radiomics, neoadjuvant treatment, treatment response

## Abstract

Objective: To explore whether the pretreatment dynamic contrast enhancement magnetic resonance imaging (DCE-MRI) and radiomics signatures were associated with pathologic complete response (pCR) to neoadjuvant therapy (NAT) in breast cancer. Method: A retrospective review of 70 patients with breast invasive carcinomas proved by biopsy between June 2017 and October 2020 (26 patients were pathological complete response, and 44 patients were non-pathological complete response). Within the pre-contrast and five post-contrast dynamic series, a total of 1037 quantitative imaging features were extracted from in each phase. Additionally, the Δfeatures (the difference between the features before and after the comparison) were used for subsequent analysis. The least absolute shrinkage and selection operator (LASSO) regression method was used to select features related to pCR, and then use these features to train multiple machine learning classifiers to predict the probability of pCR for a given patient. The area under the curve (AUC), accuracy, sensitivity, and specificity were calculated to assess the predictive performances of the radiomics model for each of the five phases of time points. Result: Among the five phases, each individual phase performed with AUCs ranging from 0.845 to 0.919 in predicting pCR. The best single phases performance was given by the 3rd phase (AUC = 0.919, sensitivity 0.885, specificity 0.864). 5 of the features have significant differences between pCR and non-pCR groups in each phase, most features reach their maximum or minimum in the 2nd or 3rd phase. Conclusion: The radiomic features extracted from each phase of pre-treatment DCE-MRI possess discriminatory power to predict tumor response.

## 1. Introduction

Neoadjuvant therapy (NAT) has been widely used in the treatment of local late breast cancer and has become an important part of comprehensive treatment of breast cancer [1,2]. It can downstage tumor, reduce the extent of surgery, and provide opportunities for breast conserving surgery [3]. Previous studies have shown that patients who underwent NAT had similar overall survival as those who received conventional adjuvant chemotherapy, even though the disease-free survival (DFS) and overall survival (OS) can be improved when patients achieve complete response to pathology (pCR) after NAT [4,5,6,7]. However, the effect of treatment varies from patient to patient due to the high heterogeneity of breast cancer, about 80% of patients respond to NAT, but only 6% of them can achieve pCR [8,9]. Therefore, the prediction of treatment response and the identification of non-responding patients may be conducive to the adjustment of treatment strategies in time to avoid ineffective chemotherapy.

Dynamic contrast enhancement magnetic resonance imaging (DCE-MRI) is increasingly being used to evaluate the NAC response because of its high sensitivity and accuracy [10,11]. It not only reflects the morphological characteristics of the lesion, determining the size and boundary of the residual tumor, but also evaluates the changes in tissue function, microenvironment characteristics and metabolism [11,12,13,14].

Radiomics is a new emerging non-invasive method that extracts high-dimensional features of image to reflect the heterogeneity of the entire tumor [15,16]. Different from traditional MR signs, the radiomics features not only reflect the signal intensity, shape, size and volume of the lesion, but also provide texture and high-order features after wavelet transformation, which can quantitatively evaluate the whole tumor area and its surrounding regions [17,18,19,20,21]. Previous studies have proved the possibility and potential of radiomics in treatment evaluation; Fan et al.’s [22] analyzed a total of 158 image features representing the morphology, texture, and background enhancement of the tumor on the pre-treatment MR images. The results showed the radiomics features could be a valuable biomarker related to the treatment response. Braman et al. [23] demonstrated that the combination used of intra-tumor and peritumoral radiomic features based on pretreatment DCE-MRI could effectively detect the pCR to NAT. Comes et al. [24] have also shown that low-level CNN features based on pre-treatment MRI have high efficacy in predicting pCR. However, most of these studies only use the features extracted from the single-phase of DCE-MRI, which only reflected the spatial heterogeneity of the tumor at that point in time. As has been well established, the enhancement pattern of the tumor will change with the scanning time; therefore, the radiomic features from the multi-phase of post-contrast-enhanced MR images may provide more information about the changes in tumor characteristics over time point.

In this study, we extracted the radiomics features from all phases of post enhancement images in DCE-MRI, and aimed to evaluate the predictive performance of different combinations of feature selection in different time points.

## 2. Materials and Methods

### 2.1. Study Patients

We conducted a retrospective review of 70 patients with breast-invasive carcinomas proven by biopsy between June 2017 and October 2020. All the patients met the following inclusion criteria: pathologically confirmed invasive carcinoma; underwent breast MRI before NAT; received at least 6 cycles of NAT; surgical resection was performed after neoadjuvant chemotherapy, and the final pathological results were obtained. Exclusion criteria: images with poor quality or severe artifacts; distant metastasis during NAT. Finally, 70 patients were included in the data analysis. All cases were female, aged 28–69 years, with an average age of 47.11 ± 9.59 years.

The clinical and histological data including age, menstrual status, histological grade, the expression status of estrogen receptor (ER), progestogen receptor (PR), HER-2 and Ki-67 were collected.

### 2.2. MR Examination

All the examinations were performed in SIEMENS Verio 3.0T MR imaging system with the patient in a prone position using a 4-channel breast surface coil. The MR imaging examination consisted of the following protocol: T2 turbo inversion recovery magnitude (TR 4300 ms, TE 61.0 ms, section thickness 4 mm, FOV 340 mm × 340 mm); axial 3D FLASH T1 (TR 6.05 ms, TE 2.46 ms, section thickness 1.3 mm, FOV 340 mm × 340 mm); T2-weighted turbo spin-echo (TR 4500 ms, TE 79 ms, section thickness 4.0 mm, FOV 340 mm × 340 mm). DCE imaging was performed with one pre-contrast and five post-contrast dynamic series using axial 3D FLASH T1 with fat suppression (TR 4.67 ms, TE 1.66 ms, section thickness 1.3 mm, FOV 360 mm × 360 mm). A 0.1-mmol/kg bolus of Gadobenate (Multihance, BRACCO, Milano) was injected using high pressure injector, then followed by a 15 mL saline flush.

### 2.3. Assessment Response to Treatment

According to Miller&Payne system patients were divided into pCR group (*n* = 26) and non-pCR group (*n* = 44). pCR was defined as the absence of residual invasive cancer with or without ductal carcinoma in situ in the breast tissue and the absence of any tumor deposits in the sampled axillary nodes.

### 2.4. Radiomic Analysis

#### 2.4.1. Tumor Segmentation

Segmentation was performed using ITK-snap (version 3.8.0-beta, Orlando, FL, USA; http://www.itksnap.org; accessed on 21 May 2021) on axial fat suppression T1-weighted images obtained in a 6-image series, including pre-contrast image and 5 phases after contrast material injection. The region of interest was manually drawn on each slice along the contour of the tumor on the first postcontrast of DCE images to get the 3D segmentation of the whole tumor. The region of interest (ROI) should cover all the tumor, including the areas of necrosis and hemorrhage, but avoid edema, blood vessels, and the normal fibroglandular tissue. Then, the 3D segmented contouring based on the 1st postcontrast phase images were propagated to pre-contrast and other four post-contrast phases of DCE images. Finally, all ROIs were reviewed by another breast radiologist (with 15 years of experience).

#### 2.4.2. Feature Extraction

Before the feature extraction, isotropic voxel was resampled into 1 mm × 1 mm × 1 mm with linear interpolation for the purpose of normalizing the geometry of MR images. The in-house platform AK software (Artificial Intelligence Kit, version 3.3.0, GE Healthcare, Shanghai, China) was used to calculate the MR image features of 6 phases (pre-contrast and five post-contrast phases), with a total of 1037 quantitative imaging features in each phase. Additionally, the bin width was set to 5 due to the characteristics of MR images according to the image biomarker standardization initiative (IBSI). The features can be classified into four groups, including shape- and size-based features, first-order statistical features, texture features and wavelet features.

**Figure 1:**
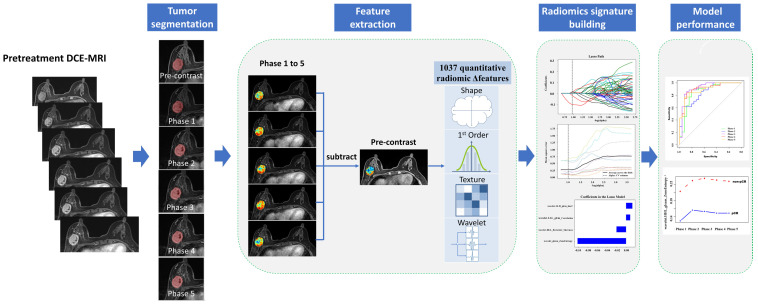
The workflow of the research.

The shape-based features were independent from the gray level intensity distribution in the image and were measured using the shape descriptors of three-dimensional size and shape of the ROI. First-order statistical features were used to describe the distribution of voxel intensities. Texture features used a matrix to represent the spatial heterogeneity of the intensity level. To further investigate the intra-ROI heterogeneity, wavelet filters were applied to the original images to convert original images to versions focused on information at different scales. wavelet decompositions with all possible combinations of high (H)- or low (L)-pass filter in each of the three dimensions (HHH, HHL, HLH, LHH, LLL, LLH, LHL, and HLL) were applied. The delta-features were calculated to represent the feature changes of each post-contrast phase and to subsequent modeling analysis (Figure 1). We use Δfeatures for the next analysis; Δfeatures were defined as the difference between the pre-contrast features and pos-contrast features, as follows:

Δfeature _*n*_ = (Feature _phase *n*_ − Feature _pre-contrast_)/Feature _pre-contrast_ (*n* = 1, 2, 3, 4, 5)

#### 2.4.3. Feature Selection

As the reproducibility of radiomic features may be influenced by the manual delineation of ROI, the 30 patients including 150 ROIs were randomly chosen to take the secondly segmentation. Additionally, we calculated the interclass and intraclass correlation coefficients (ICC) of each feature for inter-observers and intra-observers. Any features with ICC < 0.8 was considered unreliable and discarded. The z-score method was used to standardize the features to decrease the influence of dimension. To further eliminate redundant features, the Max-Relevance and Min-Redundancy (mRMR) was performed. The remaining radiomic features may still have a larger size than the sample size, which may bring the risk of overfitting. Thus, we used the least absolute shrinkage and selection operator (LASSO) to select the most predictive features, and the Akaike information criterion (AIC) was applied in the step method. Finally, logistic regression was used to construct the model which was built as the weighted sum of the selected radiomic features.

#### 2.4.4. Model Evaluation and Statistical Analysis

Models were evaluated by the ten-fold cross-validation method, which has been widely used as a reliable approach to evaluate a model’s true generalization performance. The diagnostic performance of the models was quantified by the area under the receiver operating characteristics (ROC) curve (AUC), and the 95%CI confidence interval (CI) of AUC were calculated. The optimal cutoff value was selected as the point when the sensitivity plus specificity was maximal. The sensitivity, specificity and prediction accuracy (ACC) of each model were also calculated in the cohort.

Normal distribution data are presented as mean ± standard deviation, whereas non-normally distributed data are presented as median (25–75th percentiles). The two-sample t-test or Mann–Whitney U test was used to evaluate differences in the response. A repeated measures analysis of variance was applied to assess the differences in the selected parameters at different time points in the same group, followed by the Bonferroni correlation for post hoc pairwise comparisons. All statistical analyses were performed with R software (R Foundation for Statistical Computing, Vienna, Austria; https://www.R-project.org; accessed on 16 March 2021). *p* < 0.05 was considered statistically significant. The workflow of radiomic research is shown in Figure 1.

## 3. Results

### 3.1. Patient Characteristics

The clinical characteristics of patients were shown in Table 1. There was no significant difference in age, menstrual status, histological grade, the expression status of HER-2 and Ki-67 between pCR and non-pCR group (*p* > 0.05). The number of patients with estrogen receptor (ER) positive/progesterone receptor (PR) positive in pCR group were significantly less than that in non-pCR group, and there were statistically significant differences between these two groups (*p* < 0.05). In pCR group, the largest proportion is triple negative type (34.6%), while in non-pCR group, the largest proportion is type Luminal B (63.7%). The molecular subtype distribution shows a significant difference between the two groups (*p* = 0.016).

### 3.2. Radiomics Signature Building

A total of 1037 quantitative imaging Δfeatures in each contrast phase were included in the intra-class correlation. Features with ICC < 0.8 were excluded; 485 features were selected for further analysis. Before feature selection, the abnormal or missing values were replaced by the median, and features standardization was applied. Next, the mRMR and LASSO were used to select the most optimal features. After the redundant and irrelevant features were removed by mRMR, 50 features from each ROI were retained. Then, the LASSO was applied to decrease the feature redundancy with the Akaike information criteria. After the number of features was determined, the most predictive subset of features was chosen and the corresponding coefficients were calculated.

All the Δfeatures extracted from each phase were listed Table 2. After features selection, six features on Phase_1 model, 4 features on Phase_2 model, ten features on Phase_3 model, nine features on Phase_4 model and eight features on the Phase_5 model remained in the training set independently. These features are significantly different between non-pCR and pCR groups (all *p* < 0.05).

### 3.3. Model Performance Evaluation

**Figure 2:**
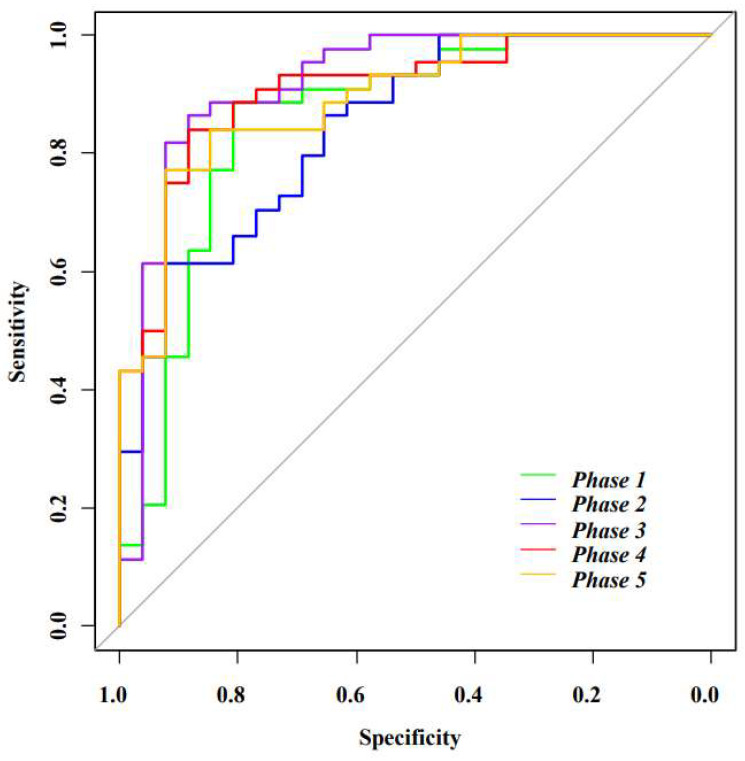
ROC curve for different phases.

The ROC analysis was used to calculate the prediction performance of different combinations of feature selection for each of the five time point phases in radiomics. AUC under ROCs for each phase were shown in Figure 2 and Table 3. The result showed each individual phase has good performance in predicting pCR with AUCs ranging from 0.845 to 0.919. The features extracted from the 3rd phase images after injection of contrast agent had the best performance among all the phases with AUC = 0.919 (sensitivity 0.885, specificity 0.864, accuracy of 0.857). This analysis suggests that the features extracted from each phase potentially possess discriminatory power to predict tumor response from the pre-treatment images.

### 3.4. Analysis of Features in Different Phases

Of all 37 features in the 1st–5th phases, there was overlap in the features selection in different phases, of which eight features were selected repeatedly in more than two phases. All these features were texture features from GLSZM GLDM GLCM and GLRLM, and half of the features underwent wavelet transformation.

Further analysis of these eight features, Table 4 showed the feature values between pCR and non-pCR groups in different time points. The results showed that one feature has different values at different time points, the feature value changes as the scanning time progresses, and most features reach their maximum or minimum in the 2nd or 3rd phase. Moreover, five of these features have significant differences between pCR and non-pCR groups in each phase and three of them were ZoneEntropy-associated descriptors.

## 4. Discussion

This study investigated the ability of machine learning model based on pretreatment multi-phase DCE-MRI to predict tumor treatment response of NAT. The results indicated that the radiomics features of the images in each post-enhancement phases have good performance for predicting pCR.

Most of the previous research on breast cancer radiomics only focused on the features extracted from one single phase after enhancement [25,26]. The prediction performance of other phases of DCE-MRI was still relatively unexplored. In this study, we made full use of all the phase in DCE-MR imaging, and analyzed the predictive abilities of the machine learning model in different phase. We found that each individual phase has good performance in predicting pCR (AUC = 0.858, 0.845, 0.919, 0.906 and 0.892). The model based on features extracted from 3rd phase image has the best prediction performance with an AUC value of 0.919. One possible explanation for this result is that the most aggressive tissue components of tumor appear to be enhanced in the early stage, while the enhancement of relatively less aggressive components gradually increase. Therefore, compared with the 90 s after the injection of the contrast agent, in the 3rd phase (about 150 s), the internal components of the tumor were enhanced more comprehensively and showed higher heterogeneity. In our study, half of the features were wavelet-transformed feature. This indicates that the wavelet transformed feature may have a high correlation with pCR in each phase. The wavelet transformation can concentrate the intensity and texture features of the original image in different frequency ranges within the tumor volume and separate images into high frequency (heterogeneity) and low frequency (homogeneity); therefore, many studies on radiomics use wavelet transform features to build predictive models. Mahrooghy et al. [27] found that the wavelet features extracted based on DCE-MRI can reflect the heterogeneity of breast cancer, and the constructed breast cancer prognosis classification model also has high predictive power. Zhou et al. [28] also indicated the inclusion of wavelet features may improve the performance of the prediction model.

Additionally, the eight features repeatedly selected in multiple phases were all texture features. ZoneEntropy, GrayLevelNonUniformity and Small DependenceEmphasis weretexture features quantifying uncertainty or randomness of the image value, and may associated with tumor heterogeneity [29]. In this study, the result in Table 4 showed that the level of texture features (e.g., ZoneEntropy, GrayLevelNonUniformity, Small DependenceEmphasis) in pCR group was significantly lower than that in non-pCR group. Prior studies demonstrated that tumors in the non-PCR group were found to contain necrosis, neovascularization, and sclerosis in addition to intermingled tumor cells, which resulted in mixed internal components of the lesion, so it was more likely to appear as heterogeneous in the image [6,30,31]. Thus, the features which related to image heterogeneity expressed more strongly within the region of non-responders during each enhancement phase in pretreatment images, and may be associated with poor prognosis. Previous studies also confirmed the utility of texture features in predicting the pathological response of breast cancer to neoadjuvant chemotherapy. Braman et al. [23] analyzed pretreatment breast DCE-MRI images and found that the combination use of intratumoral and peritumoral texture features could predicting pCR to neoadjuvant chemotherapy. Chamming’S et al. [18] also indicated that the texture features extracted from pretreatment MR image could distinguish the molecular subtypes of breast cancer.

There are still deficiencies in this study. This study is based on a limited number of patients. A larger sample size is needed to verify the validity and practicability of these classifiers. Additionally, we only analyzed the T1-weighted image after contrast material injection, T2-weighted image, DWI and other multiple sequences need to be analyzed in the later stage of the study to construct a multimodal MR radiomics model.

## 5. Conclusions

The delta radiomic features extract from pre-treatment DCE-MRI could be the predictor of NAT response, and the radiomics model constructed in our study can effectively predict pCR.

## Figures and Tables

**Table 1:** Patient Characteristics.

Variable	pCR (*n* = 26)	non-pCR (*n* = 44)	*p*-Value
Age (Mean years ± SD)	47.55 ± 10.29	46.92 ± 9.36	0.801
Menstrual status			0.939
Postmenopausal	13 (50.0)	21 (49.0)	
Premenopausal	13 (50.0)	23 (51.0)	
Histological type			1.000
IDC	26 (100)	43 (98.0)	
ILC	0 (0.0)	1 (2.0)	
Histologic grade			0.097
2	9 (34.6)	16 (36.4)	
3	17 (65.4)	28 (63.6)	
Molecular subtype			0.016 *
Luminal A	2 (7.7)	7 (15.9)	
Luminal B	7 (26.9)	28 (63.7)	
HER-2 enriched	8 (30.8)	3 (6.8)	
TNBC	9 (34.6)	6 (13.6)	
ER			0.004 *
Positive	9 (34.6)	31 (70.5)	
Negative	17 (65.4)	13 (29.5)	
PR			0.001 *
Positive	5 (19.2)	29 (65.9)	
Negative	21 (80.8)	15 (34.1)	
HER-2			0.734
Positive	12 (46.2)	18 (40.9)	
Negative	14 (53.8)	26 (59.1)	
Ki 67			0.092
<14%	5 (19.2)	12 (27.3)	
≥14%	21 (80.8)	32 (72.7)	

* *p* < 0.05; estrogen receptor (ER), progesterone receptor (PR), human epidermal growth factor receptor 2 (HER-2), triple negative breast cancer (TNBC).

**Table 2:** Radiomic features for each time point.

Phase 1	wavelet.LLH_glszm_ZoneEntropy
	log.sigma.3.0.mm.3D_gldm_SmallDependenceEmphasis
	wavelet.HHL_glszm_ZoneEntropy
	log.sigma.3.0.mm.3D_glszm_GrayLevelNonUniformity
	wavelet.LHL_gldm_SmallDependenceLowGrayLevelEmphasis
	original_gldm_LargeDependenceHighGrayLevelEmphasis
Phase 2	wavelet.HHL_glszm_ZoneEntropy
	log.sigma.3.0.mm.3D_gldm_SmallDependenceEmphasis
	original_glszm_GrayLevelNonUniformity
	wavelet.HLL_glrlm_LongRunHighGrayLevelEmphasis
Phase 3	original_shape_Maximum2DDiameterSlice
	wavelet.LLH_glszm_ZoneEntropy
	wavelet.HHL_glszm_ZoneEntropy
	original_glszm_GrayLevelNonUniformity
	log.sigma.2.0.mm.3D_glcm_ClusterShade
	original_gldm_LargeDependenceHighGrayLevelEmphasis
	log.sigma.3.0.mm.3D_gldm_SmallDependenceEmphasis
	wavelet.HLL_glrlm_LongRunHighGrayLevelEmphasis
	log.sigma.2.0.mm.3D_glrlm_LongRunHighGrayLevelEmphasis
	wavelet.LLH_firstorder_Median
Phase 4	wavelet.LLH_glszm_ZoneEntropy
	original_glszm_GrayLevelNonUniformity
	wavelet.HLH_glszm_ZoneEntropy
	wavelet.HHL_glszm_ZoneEntropy
	log.sigma.2.0.mm.3D_glcm_ClusterShade
	wavelet.LLL_glcm_Correlation
	log.sigma.3.0.mm.3D_glszm_GrayLevelNonUniformity
	wavelet.LLH_gldm_SmallDependenceLowGrayLevelEmphasis
	original_gldm_LargeDependenceHighGrayLevelEmphasis
Phase 5	wavelet.LLH_glszm_ZoneEntropy
	original_glszm_GrayLevelNonUniformity
	wavelet.HHL_glszm_ZoneEntropy
	wavelet.LLL_glcm_Correlation
	wavelet.HLH_glszm_ZoneEntropy
	log.sigma.2.0.mm.3D_glcm_ClusterShade
	log.sigma.3.0.mm.3D_glszm_GrayLevelNonUniformity
	log.sigma.2.0.mm.3D_glrlm_LongRunHighGrayLevelEmphasis

**Table 3:** Comparison of predictive performance of six models in the cohort.

Model	AUC (95%CI)	Sensitivity	Specificity	Accuracy	Youden Index
Phase 1	0.858(0.757–0.959)	0.886	0.808	0.786	0.694
Phase 2	0.845 (0.753–0.938)	0.614	0.923	0.771	0.537
Phase 3	0.919 (0.842–0.996)	0.864	0.885	0.857	0.748
Phase 4	0.906 (0.835–0.978)	0.841	0.885	0.843	0.726
Phase 5	0.892 (0.815–0.968)	0.773	0.923	0.786	0.696

**Table 4:** The selected Δfeatures in the non-pCR and pCR groups at each time point.

Time Point	non-pCR (*n* = 26)	pCR (*n* = 44)	Statistics	*p*-Value
wavelet.LLH_glszm_ZoneEntropy				
Phase 1	0.19 (0.13, 0.24)	0.13 (0.08, 0.16)	3.257	0.001 *
Phase 2	0.19 (0.17, 0.26)	0.14 (0.10, 0.21)	3.124	0.002 *
Phase 3	0.20(0.17, 0.27)	0.13(0.10, 0.20)	3.343	0.001 *
Phase 4	0.19(0.17, 0.26)	0.13(0.09, 0.21)	3.367	0.001 *
Phase 5	0.20(0.16, 0.26)	0.13(0.10, 0.20)	3.379	0.001 *
original_glszm_GrayLevelNonUniformity				
Phase 1	1.41(0.71, 2.55)	0.87(0.53, 1.69)	2.018	0.044 *
Phase 2	2.32 ± 1.20	1.60 ± 0.91	2.837	0.006 *
Phase 3	2.41 ± 1.09	1.64 ± 0.83	3.096	0.003 *
Phase 4	2.26 ± 0.99	1.60 ± 0.79	3.071	0.003 *
Phase 5	2.23 ± 0.91	1.55 ± 0.76	3.358	0.001 *
log.sigma.3.0.mm.3D_gldm_ SmallDependenceEmphasis				
Phase 1	2.08 ± 1.19	1.44 ± 0.90	2.578	0.012 *
Phase 2	3.01(2.54, 3.62)	2.59(1.77, 2.84)	2.723	0.006 *
Phase 3	3.14(2.35, 3.57)	2.54(1.99, 2.93)	2.443	0.015 *
Phase 4	2.93(2.17, 3.58)	2.39(1.88, 2.90)	2.261	0.024 *
Phase 5	2.88 ± 1.02	2.31 ± 0.88	2.502	0.015 *
wavelet.LLL_glcm_Correlation				
Phase 1	0.03 ± 0.07	0.05 ± 0.08	−1.033	0.305
Phase 2	0.01 ± 0.08	0.03 ± 0.08	−1.342	0.184
Phase 3	−0.02 ± 0.08	0.02 ± 0.09	−1.997	0.05 *
Phase 4	−0.01(−0.09, 0.05)	0.01(−0.01, 0.07)	−1.92	0.055
Phase 5	−0.02(−0.09, 0.03)	0.02(−0.01, 0.05)	−2.601	0.009 *
wavelet.HHL_glszm_ZoneEntropy				
Phase 1	0.42(0.11, 0.71)	0.18(0.06, 0.33)	2.686	0.007 *
Phase 2	0.47(0.20, 0.80)	0.22(0.08, 0.39)	3.051	0.002 *
Phase 3	0.50(0.20, 0.87)	0.20(0.09, 0.36)	3.33	0.001 *
Phase 4	0.49(0.17, 0.70)	0.23(0.09, 0.35)	2.662	0.008 *
Phase 5	0.46(0.20, 0.78)	0.23(0.10, 0.33)	3.257	0.001 *
log.sigma.2.0.mm.3D_glcm_ ClusterShade				
Phase 1	−5.36(−31.94, 0.52)	1.98(−2.03, 26.89)	−3.233	0.001 *
Phase 2	−4.11(−33.18, 6.39)	3.39(−7.09, 60.64)	−2.127	0.033 *
Phase 3	−5.72(−30.67, 7.14)	3.97(−6.00, 55.44)	−2.37	0.018 *
Phase 4	0.25(−31.26, 9.41)	3.71(−5.23, 61.37)	−1.993	0.046 *
Phase 5	0.02(−29.43, 11.50)	6.43(−3.48, 56.29)	−1.872	0.061
log.sigma.2.0.mm.3D_glrlm_ LongRunHighGrayLevelEmphasis				
Phase 1	0.20(−0.12, 0.45)	−0.02(−0.23, 0.26)	1.155	0.248
Phase 2	0.27 ± 0.57	0.10 ± 0.44	1.378	0.173
Phase 3	0.33 ± 0.58	0.08 ± 0.43	2.102	0.039 *
Phase 4	0.34 ± 0.61	0.08 ± 0.42	1.935	0.06
Phase 5	0.28(−0.20, 0.68)	0.00(−0.30, 0.31)	1.69	0.091
wavelet.HLH_glszm_ZoneEntropy				
Phase 1	0.37(0.19, 0.61)	0.16(0.06, 0.35)	2.565	0.01 *
Phase 2	0.39(0.26, 0.63)	0.24(0.12, 0.39)	2.82	0.005 *
Phase 3	0.41(0.25, 0.65)	0.23(0.12, 0.39)	2.929	0.003 *
Phase 4	0.43(0.23, 0.68)	0.23(0.13, 0.34)	2.808	0.005 *
Phase 5	0.42(0.22, 0.67)	0.24(0.11, 0.37)	2.893	0.004 *

* *p* < 0.05.

## Data Availability

The datasets generated during and/or analyzed during the current study are available from the corresponding author on reasonable request.
